# MULTILEVEL PERCEPTIONS OF IMPLEMENTING SHARING CHOICES: A QUALITATIVE STUDY

**DOI:** 10.1093/geroni/igad104.1529

**Published:** 2023-12-21

**Authors:** Martha Abshire Saylor, Valecia Hanna, Sydney Dy, Danetta Sloan

**Affiliations:** Johns Hopkins University School of Nursing, Baltimore, Maryland, United States; Johns Hopkins School of Public Health, Baltimore, Maryland, United States; Johns Hopkins School of Public Health, Baltimore, Maryland, United States; Johns Hopkins Bloomberg School of Public Health, Baltimore, Maryland, United States

## Abstract

Reviews have found that ACP interventions alone, including those in primary care, improve advance directive documentation but have not shown effectiveness for person-centered or end-of-life outcomes. One potential reason is that these interventions may not be sufficiently comprehensive and do not address the wide scope of population, communication and systems issues needed to improve outcomes. We conducted semi-structured interviews to explore experiences with the SHARING Choices intervention through the perspectives of key stakeholders involved in the trial. We recruited all embedded ACP facilitators; the clinicians and practice managers from primary care practices from both participating health systems; and patients (and/or family) who participated in the intervention. Tailored interview guides addressed how implementation occurred and evolved, acceptability and key enablers/barriers/adjustments in implementation, and the use and perceived impact of different elements of the intervention. A multidisciplinary team generated a coding scheme based on these topics of interest and used thematic analysis to identify, analyze, describe, and report emergent themes from responses across interview groups. We interviewed 40 participants (all 9 facilitators, 11 patients and families, and 20 clinicians and administrators). We will present major themes and differences across interview groups, and how this informs the quantitative results of the pragmatic trial.

